# Prospective Non-Randomized Study of Intraoperative Assessment of Surgical Resection Margin of Colo-Rectal Liver Metastases

**DOI:** 10.7150/jca.58580

**Published:** 2021-04-30

**Authors:** Mladjan Protic, Olivera Krsmanovic, Nenad Solajic, Biljana Kukic, Ivan Nikolic, Bogdan Bogdanovic, Zoran Radovanovic, Milana Kresoja, Ciaran Mannion, Yan-Gao Man, Alexander Stojadinovic

**Affiliations:** 1 Clinic for Surgical Oncology, Oncology Institute of Vojvodina, Sremska Kamenica, Serbia.; 2 Faculty of Medicine, University of Novi Sad, Novi Sad, Serbia.; 3 Armed Forces of Bosnia and Herzegovina, Logistics Command, Doboj, Bosnia and Herzegovina.; 4 Department of Pathoanatomical and Laboratory Diagnostics, Oncology Institute of Vojvodina, Sremska Kamenica, Serbia.; 5 Clinic for Internal Oncology, Oncology Institute of Vojvodina, Sremska Kamenica, Serbia.; 6 Department of Pathology, Hackensack University Medical Center, Hackensack, New Jersey, USA.; 7 Department of Pathology, Hackensack Meridian School of Medicine, Nutley, New Jersey, USA.; 8 Uniformed Services University of the Health Sciences, Bethesda, Maryland, USA.

**Keywords:** colon cancer, liver metastases, resection, resection margin, recurrence, disease-free survival

## Abstract

**Introduction:** More than 50% of patients with colorectal cancer (CRC) develop liver metastases during the natural course of disease. Surgical resection is currently the most potentially curative method in the treatment of colorectal liver metastases (CRLM). The goal of surgery is to achieve a negative resection margin (RM) of at least 1 mm, which provides the best prognosis for patients. The RM can be assessed by the pathologist of the resected liver specimen (RLS) and by the surgeon intraoperatively. The aim of this research paper is to determine the degree of agreement on intraoperative assessment of the RM by the surgeon and histopathological RM assessment by the pathologist.

**Material and methods:** This prospective non-randomized double-blind study was approved by the Ethics Committee of the Oncology Institute of Vojvodina and registered on ClinicalTrials.gov #NCT04634526. The study was conducted at the Oncology Institute of Vojvodina, Sremska Kamenica, Serbia. An experienced hepatobiliary surgeon assessed RM for every specimen intra-operatively, immediately after CRLM resection. Resected CRLM lesions were analyzed by two experienced pathologists. These data were compared with pathological RM assessment as a “gold standard”. RM of 1 mm or more was rated as negative RM (RM-). Disease-free survival (DFS) and recurrence rate was calculated by RM status defined by surgeon and by pathologist.

**Results:** From 01 January 2015 to 31 August 2019, 98 patients were enrolled in the study. There were 219 RLS with 245 CRLM. The surgeon registered positive RM (RM+) of <1mm in 41 (18.7%) RLS. Taking the result of the histopathological assessment (HPA) as the “gold standard”, it was determined that RM was true positive in 32 (14.6%) cases. False positive RM was found in 9 (4.1%) cases. False negative RM was found in 20 (9.1%) cases. True negative RM was found in 158 (72.2%) cases. Sensitivity of surgical assessment (SA) of RM+ was 61.5% (32/52). Specificity of SA of RM+ was 94.6% (158/167). The positive predictive value (PPV) was 78.0% (32/41), while the negative predictive value (NPV) was 88.8% (158/178). The overall accuracy of the RM+ SA was 86.8% (190/219). There was no statistically significant difference in the assessment of RM+ per RLS by surgeon and pathologists (p=0.061), but it was significant when analyses per patients was performed (p=0.017). Recurrence rate for RM+ patients was 48.1% (13/27, p=0.05) for SA and 35.0% (14/40, p=0.17) for HPA. Three year DFS for RM- and RM+ was 66.5% and 27.9% (p=0.04), respectively, by SA, and 64.8% and 42.1% (p=0.106), respectively, by HPA.

**Conclusion:** Intraoperative assessment of RM- by surgeon of RLS is clinically meaningful. There is not a statistically significant difference in the assessment of RM+ by surgeon and pathologists per RLS, but it was statically significant on a per patient basis. RM determined by surgeon has better prognostic impact on recurrence rate and 1- and 3-year DFS than standard histopathological assessment.

## Introduction

Colorectal cancer (CRC) is the second leading cause of cancer mortality worldwide, with 1.8 million new cases and 818,000 estimated deaths in 2018 1. These CRC-related deaths occurred in most cases due to occurrence of CRC metastases. The liver is the most common site of CRC metastasis and ~50% of patients with CRC develop liver metastases during the natural course of disease; hence, CRLM represents a serious health problem today 1-3. The most meaningful way to treat patients with CRLM, that provides a potentially curative effect, is complete resection of the liver metastases 4-13. The status of the CRLM resection margin is an established prognostic factor, and the goal of surgical treatment is to achieve a negative resection margin 14. From the initially proposed 10 mm RM 15-17, the width of the free edge has decreased with research insights gained over time 18-20. De Haas began the era of 'safe free edge' of 1 mm 21. Today, most authors believe that a RM of 1 mm free edge is sufficient to achieve a curative effect 22-28. Possible exceptions could be cases in which the metastasis is located adjacent to the wall of a blood vessel, but does not infiltrate it, such that a free edge of 0 mm could be considered a negative RM in this case 29. However, there are also authors who claim that a free edge of 0 mm may be sufficient to define negative RM 30, 31. Many authors believe that achieving RM- remains an important therapeutic determinant and should be the primary goal of surgical treatment, while RM+ increases the risk of disease relapse 28, which leads to decreased disease-free and overall survival (OS) 31-35. On the other hand, there is a group of authors who emphasize the positive effect of modern systemic therapy on DFS and OS, and suggest that RM+ status after CRLM resection may not have the prognostic significance for patients with good response to neoadjuvant chemotherapy with or without additional biological therapy 36-39.

There are tools utilized to define the neoplastic lesion and to determine RM using different optical methods, but intra-operative ultrasonography (IOUS), palpation and visualization remain the standard of care (SOC) for intra-operative detection of the liver lesions and assessment of RM 40-45. The assessment of RM can be performed by the surgeon intra-operatively and by the pathologist on the resected liver specimen. In all previous studies, the results were presented based on the assessment of RM by a pathologist, and the assessment of RM by the pathologist is recognized as SOC.

In the prognostic sense, it is important that RM assessment by surgeon and pathologist agree as much as possible. However, in clinical practice we see that there is also the possibility of disagreement, as the surgeon does not know RM status at a microscopic level, while the pathologist examines only the RLS and does not know what remains in the patient's liver. Also, it is unknown precisely what effect utilization of energy devices during liver parenchymal transection has on RM and clinical outcome.

A literature search in this specific area of CRC research (Web of Science for key words “colorectal liver metastases” and “resection margin”) did not reveal that anyone has thus far investigated the clinical significance of standard of care intraoperative assessment of RM for CRLM. Moreover, the literature search yielded no result for investigations comparing standard intraoperative RM of CRLM assessment to histopathological examination as the “gold standard”. The aim of this study is to assess the rate of agreement and diagnostic value of RM standard assessed by surgeon and pathologist, and its clinical significance.

## Material and methods

### Patients

This is a prospective, single arm, non-randomized double blinded study. It was conducted at the Institute of Oncology of Vojvodina, Sremska Kamenica, Serbia. The research was approved by the Ethics Committee of the Institute of Oncology of Vojvodina, Sremska Kamenica, Serbia (No. 4/18/1-972-9) and it is registered on ClinicalTrials.gov #NCT04634526.

All patients were first informed of the objectives of the study after which they voluntarily provided informed consent to participate in this clinical study.

**Inclusion criteria were:**

- Age of patients from 18 to 85 years;
- Preoperative diagnosis of CRLM;
- Indication for surgical resection of CRLM;
- Defined status of the resection margin by the surgeon immediately after resection;
- Defined status of the resection margin by the pathologist after examination of the RLS.

**Exclusion criteria were:**

- RM was not defined by surgeon and/or pathologist as positive or negative;
- Surgical resection and application of ablative procedures at the same time;
- Previous liver surgery for CRLM;
- Concomitant use of pre-operative neoadjuvant chemotherapy was not a reason to exclude patients from the study.

### Pre-operative evaluation

In all patients, the pre-operative diagnosis of liver metastases of colorectal adenocarcinoma origin was established by contrast-enhanced computed tomography (CT) and or magnetic resonance imaging (MRI). The patients were presented to the Multi-Disciplinary Team (MDT) for hepato-biliary-pancreatic (HPB) diseases, which include at a minimum in attendance: HPB surgeon, medical oncologist, and radiologist. During the analysis of imaging (CT and/or MRI) findings, the resectability of the present hepatic lesions was assessed and further treatment needed was determined. The size of the largest CRLM lesion was also recorded. Patients with small solitary lesions and those diagnosed 2 or more year after operation of primary tumor were sent to up-front surgery. For other patients chemotherapy was indicated (doublet, FOLFOX4 or FOLFIRI). If the CRLM lesion was deemed not operable at the time of its diagnosis, conversion therapy was included, doublet chemotherapy with biological therapy (bevacizumab or cetuximab). After its implementation, the therapeutic response was assessed by MDT according to the Response Evaluation Criteria in Solid Tumors (RECIST) Version 1.1 criteria. Possible RECIST v1.1 therapeutic response criteria for target lesions includes complete response (CR), partial response (PR), stable disease (SD) and progressive disease (PD). Surgical resection was indicated in the cases with resectable disease in the absence of PD on neoadjuvant therapy and general contraindication.

Synchronous metastases are metastatic lesions present in the liver at time of diagnosis of the primary colorectal tumor or for a maximum of 3 months after diagnosis of the primary tumor. If a metastatic lesion was diagnosed >3 months after the diagnosis of the primary tumor, then it was defined as metachronous CRLM.

All patients enrolled in the study underwent CRLM surgery for the first time. In patients with metachronous CRLM in whom bowel surgery was performed before surgery of CRLM, data for the primary tumor and initial treatment intervention were obtain from previous medical history and medical record reports, while the assessment of RM was performed prospectively.

### Surgery

In this study all liver resections were performed by one experienced HPB surgeon who performs >50 liver resections per year. The surgery was performed by open or laparoscopic approach under general endotracheal anesthesia. CRLMs were identified intra-operatively visually, by palpation, and by IOUS (Ultrasound scanner 1202, BK medical) for each patient after mobilization of the liver. Technique of parenchymal transection was not pre-defined in this study and crush and clamp, harmonic scalpel (Ethicon Endo-Surgery) and hydro-jet (ERBEJET 2) was used according to the surgeon decision. Electrocoagulation could be used to treat remaining resected liver surface to achieve hemostasis.

The CRLM resection margin was assessed intra-operatively by inspection and palpation of the resection margin for each RLS. If there was no whitish hard tissue at the site of the removed metastatic lesion and if mobility of the surrounding liver parenchyma was present, the resection margin was assessed as negative. If the surgical incision was performed on the surface induration itself, and there were no traces of induration left on the remaining liver parenchyma, then the surgeon assessed the resection margin as R1. When, after removal of metastatic lesions in the liver parenchyma, there was a remaining hard consistency tissue that was immobile or less mobile than the rest of the liver, or a complete metastatic lesion remained, the RM was rated as R2. We marked the R1 and R2 resection margins as positive (RM+) and R2 resection was assigned as 'incomplete resection'. In the case of multiple CRLM lesions, the RM per patient was determined to be 'positive' when at least one of the lesions had a positive RM.

### Pathological examination

The resection margin was assessed by hematoxylin and eosin (H&E)-stained slides by two experienced pathologists. The pathologists determined the number and size of metastatic lesion(s), as well as the histopathological status of the CRLM resection margin.

All resected specimens were submitted to the pathology laboratory in the fresh state. Frozen section analysis was performed when required. Surgical margins were inked circumferentially. Immediately after inking, the surgically resected specimens were immersed in 10% buffered formalin and left for fixation for 24 hours prior to gross examination. Fixed liver specimens were serially cut perpendicular to the resection margin. Representative tissue blocks of metastatic tumor and of all but obviously uninvolved (≥5 mm) margins were taken for standard histopathological processing and analysis. Microscopic examination of tissue samples was performed on 4 µm-thick slides stained routinely with H&E. No ancillary pathological or immunohistochemical techniques were used.

Classification of CRLM resection margins status was as follows: R0 - no identifiable tumor cells on or within 1 mm from the inked resection margin; R1- presence of tumor cells on the margin or within 1 mm of the margin; and, R2 - grossly visible tumor on the resection margin. Positive RM (RM+) are cases with R1 or R2 resection, but R2 cases are deemed as incompletely resected. Histologically measured distance in millimeters from tumor to the closest surgical margin was routinely reported.

Response to neoadjuvant treatment was also evaluated. In the absence of a universally accepted scoring system, we estimated proportion of infarct-like necrosis and fibrosis within the histologically examined metastatic tumor. We excluded so-called “dirty necrosis,” which is generally present in colorectal adenocarcinomas; the proportion was recorded as a percentage in 5%-increments.

### Follow up

Patients were followed after surgical recovery by the oncology team. Physical examination of the patient and analysis of serum CEA levels were performed every 3-6 months for up to 2 years, and then every 6 months for up to 5 years. Imaging (CT or MRI) of the chest, abdomen, and pelvis was performed every 3-6 months for up to 2 years, and then every 6-12 months for up to 5 years. Colonoscopy was performed after 3 years post primary tumor resection, and then every 5 years. Recurrence and/or progression was diagnosed by imaging.

### Objectives

True positive (TP) value was defined when surgeon and pathologist agreed that the RM was positive. True negative (TN) value was defined when surgeon and pathologist agreed that the RM was negative. False positive (FP) value was defined when surgeon assigned as RM+ but pathologist as RM-. False negative (FN) value was defined when surgeon assigned as RM- but pathologist as RM+. The sensitivity (Sn) of the surgical assessment of RM was defined as the proportion of the number of detected TP surgeon assessed RM and the number of detected RM+ by standard histopathological examination. The specificity (Sp) of the surgical assessment of RM was defined as the proportion of the number of detected TN surgeon assessed RM and the total number of detected RM - by the pathologist.

A positive predictive value (PPV) is defined by the proportion of the total number of detected RM+ by surgeon assessment and the number of detected RM+ summary by the pathologist and the surgeon. The negative predictive value (NPV) is defined by the proportion of the total number of RM- by surgeon assessment and the summary number of RM- assessed by the surgeon and the pathologist. The overall accuracy of the surgeon assessed RM+ is defined by the number of RLS or patients in which the surgeon and the pathologist agreed on the assessment of CRLM resection margin and the total number of RLS or patients.

### Statistics

The sample size was calculated according to the formula for determining the difference between the two proportions, for a confidence level of 95%, statistical power of the study of 80%, proportion of surgical assessment of RM+ of 10% and proportion of RM+ assessment by pathologist of 20%. The minimum sample size required is 219 RLS. The agreement of the distributions of the variables with the normal distribution was tested by Kolmogorov's Smirnov test. Student's t-test, Men's Whitney test, Wilcoxon's test, Gehan's test as well as the chi-square test were used in the statistical analysis. Equality of proportions was tested. The degree of agreement between the assessment of RM by surgeon and pathologist was assessed by the Kappa test and significance by McNemar test. Rate of agreement between surgeon and pathologist findings was determined by Cohen's kappa levels of agreement 46. Kaplan Maier test was used for calculation of time-dependent variables. Differences for which the p value was 0.05 or less were taken as statistically significant. For statistical data processing, Microsoft Excel 2007, and statistical package Statistica 13.5 (StatSoft Inc., Tulsa, OK, USA) university license for the University of Novi Sad, were used.

## Results

### Characteristics of the patients

From January 1, 2015 to August 31, 2019, 98 patients with CRLM were enrolled in the study. A total of 245 CRLMs were surgically resected in 219 RLS.

Of the total number of patients, 61 (62.2%) were men. The average age at the time of CRLM diagnosis was 62.3 ± 10.1 years, ranging from 23 to 78 years. Regarding the localization of the primary tumor, 19 (19.4%) patients had a tumor of the right colon. Synchronous metastases were present in 48 (49.0%) patients. As many as 85 (86.7%) patients had pT3 and pT4 primary tumor stage. Regional lymph nodes were negative in 26 (26.5%) patients. Lympho-vascular invasion (LVI) was present in 68 (69.4%) and peri-neural invasion (PNI) in 38 (38.8%) patients. Sixty-five (67.7%) patients had at least one positive lymph node. Nineteen (19.4%) patients had urgent primary tumor surgery. In all 19 patients, urgent surgery of the primary tumor was undertaken for colonic obstruction. In addition, two (10.5%) of these 19 patients also had colonic perforation. In terms of all these clinical and pathological parameters, we did not find a significant difference in relation to the status of RM, both when RM was assessed by a surgeon, and when it was by a pathologist. Detailed data stratified by RM determined by surgeon and pathologist are shown in **Table 1**.

### Preoperative evaluation

On the pre-operative imaging (CT or MRI), when RM was assessed by the surgeon, the diameter of the largest metastasis in the case of RM- was 29.1 ± 17.9 mm, while in the case of RM + this value was 39.4 ± 22.6 mm (p=0.055). When RM was assessed by a pathologist, the ratio of the diameter of the largest metastasis to RM- and RM + was 29.3 ± 18.8 and 35.5 ± 20.6 mm, respectively (p=0.201).

### Surgical resection

Parenchymal liver transections were done with combined 'clamp and crush' technique plus ultracision harmonic scalpel in 68 (69.4%) cases, ultracision alone in 29 (29.6%), and hydro-jet in 1 (1.0%) case. There was no statistically significant difference in RM status according to technique of parenchymal transection. The Pringle maneuver to clamp the hepatoduodenal ligament was used in 36 (36.7%) cases without statistically significant impact on RM status. Anatomical liver resection was performed in 55 (56.1%) patients, and there is no statistical difference in distribution according to RM assessed by surgeon and pathologist. Positive RM was detected in 41 (18.7%) RLS, and in 17 (26.5%) of the patients. Sixteen (16.3%) patients had liver re-resection due to CRLM recurrence. Fourteen (14.3%) patients underwent re-operation once; 1 (1.0%) patient was re-operated twice, and 1 (1.0%) three times during follow-up period. In patients who underwent multiple operations, 6 (37.5%) had an RM + resection according to the surgeon's assessment at the first operation, while 10 (62.5%) had an RM- resection. According to the pathologist, 7 (43.8%) patients had an RM + resection during the first operation.

Ninety-five (97.0%) patients underwent open and 3 (3.0%) laparoscopic surgery for resection of CRLM. All laparoscopically operated patients were assessed by surgeon and pathologist as RM-. In 70 (71.4%) cases, liver resection was performed after resection of primary tumor, in 26 (26.5%) cases simultaneous resection of the liver metastases and primary tumor and in 2 (2.04%) cases liver first approach was performed.

### Chemotherapy

Eighty-five patients (86.7%) received chemotherapy during CRLM treatment. Of these, 32 (32.7%) patients received pre-operative, 45 (45.9%) peri-operative, and 8 (8.2%) post-operative chemotherapy, alone. The average number of cycles of pre-operative chemotherapy was 10.1±4.45, with a range of 4 to 23 and a median of 10 cycles. In 19 (59.4%) patients, biological therapy was applied pre-operatively in addition to chemotherapy. The average number of cycles of peri-operative chemotherapy was 13.6±4.03 with a range of 6 to 22 cycles and a median of 12 cycles. Of the total number of patients who received peri-operative chemotherapy 26 (57.8%) received biological therapy in addition to cytotoxic chemotherapy. The average number of cycles of applied post-operative chemotherapy was 5.8±3.22, with a range from 1 to 12 and a median of 6. The number of cycles of applied post-operative chemotherapy with and without the addition of biological therapy, administered in accordance with the status of CRLM resection margin as determined by the assessment of surgeon and pathologist, are shown in **Table 1**.

### Time intervals

There was no significant difference in time interval from diagnosis of primary tumor to diagnosis of CRLM when RM was assessed by surgeon (RM- 11.0±17.9 months; RM+ 6.4±11.6 months; p=0.221) or by pathologist (RM- 11.4±19.1 months; RM+ 7.2±11.0 months; p=0.221).

There was no significant difference in time interval from diagnosis to CRLM surgery in relation to the achieved RM when assessed by surgeon (RM- 6.2± 4.79 months; RM+ 8.2±6.09 months; p=0.094) or by a pathologist (RM- 6.8±6.09 months; RM+ 6.6±5.87 months; p=0.858).

The third time interval analyzed in relation to the achieved RM was the time from primary tumor surgery to CRLM surgery: surgeon assessed RM- 14.8 ± 17.9 months vs. RM+ 12.9 ± 17.2 months; p=0.632. Pathologist assessed RM by time interval from primary tumor surgery to CRLM surgery was as follows: RM- 15.8 ± 19.3 months vs. 11.9 ± 14.7 months; p=0.292.

### Comparison of surgeon and pathologist assessed liver resection margin

#### Comparison per resected liver specimen

The results obtained by comparing the surgeon and pathologist assessed RM+ by RLS are shown in **Table 2**. True positive result was found in 32 (14.6%) cases. True negative result was found in 158 (72.2%) cases. False positive result was found in 9 (4.1%) cases. False negative result was found in 20 (9.1%) cases. Sensitivity of surgical assessment of RM+ was 61.5% (32/52). Specificity of surgical assessment of RM+ was 94.6% (158/167). Positive predictive value was 78.0% (32/41). Negative predictive value was 88.8% (158/178). Overall accuracy was 86.8% (191/219). Cohen kappa test was 0.606 consistent with moderate level of agreement between surgeon assessed and pathologist assessed RM. McNemar test was 4.17 (p=0.061).

#### Comparison per patients

The results obtained by comparing the surgeon and pathologist assessed RM+ by patients are shown in **Table 3**. True positive result was determined in 21 (21.4%) cases. False positive result was found in 5 (5.1%) cases. True negative was found in 55 (56.1%) cases. False negative result was found in 17 (17.3%) cases. Sensitivity of surgical assessment of RM+ of 55.3% (21/38) was obtained. Specificity of surgical assessment of RM+ was 91.7% (55/60). Positive predictive value was 80.8% (21/26). Negative predictive value was 88.8% (55/72). Overall accuracy was 77.6% (76/98). Cohen kappa test was 0.498 consistent with moderate level of agreement between surgeon assessed and pathologist assessed RM. McNemar test shown statistically significant distribution (p=0.017).

### Disease recurrence

Over a median follow-up period of 16.7 months (range 1 to 51 months), disease recurred in 32 (32.7%) patients, of which 27 (84.4%) had liver-only recurrence, 8 (25.0%) had synchronous liver and lung metastases, two (2.0%) had local recurrence at site of primary tumor resection, 2 (2.0%) had lung-only recurrence, and one (1.0%) patient had intra-abdominal nodal recurrence. Disease recurrences by surgeon assessed RM were 22.1% (17/77) for RM- and 57.7% (15/26) for RM+ (p=0.01). When RM was assessed by pathologist, disease recurrence by margin status was 26.7% (16/60) for RM- and 42.1% (16/38) for RM+ (p=0.115). Recurrences of disease in the liver according to the surgeon assessed RM were 22.9% (14/61) for RM- and 48.1% (13/27) for RM+ (p=0.05). Recurrences of disease in the liver according to the pathologist assessed RM were 22.4% (13/58) for RM- and 35.0% (14/40) for RM+ (p=0.17).

### Disease-free survival

**Figure 1:**
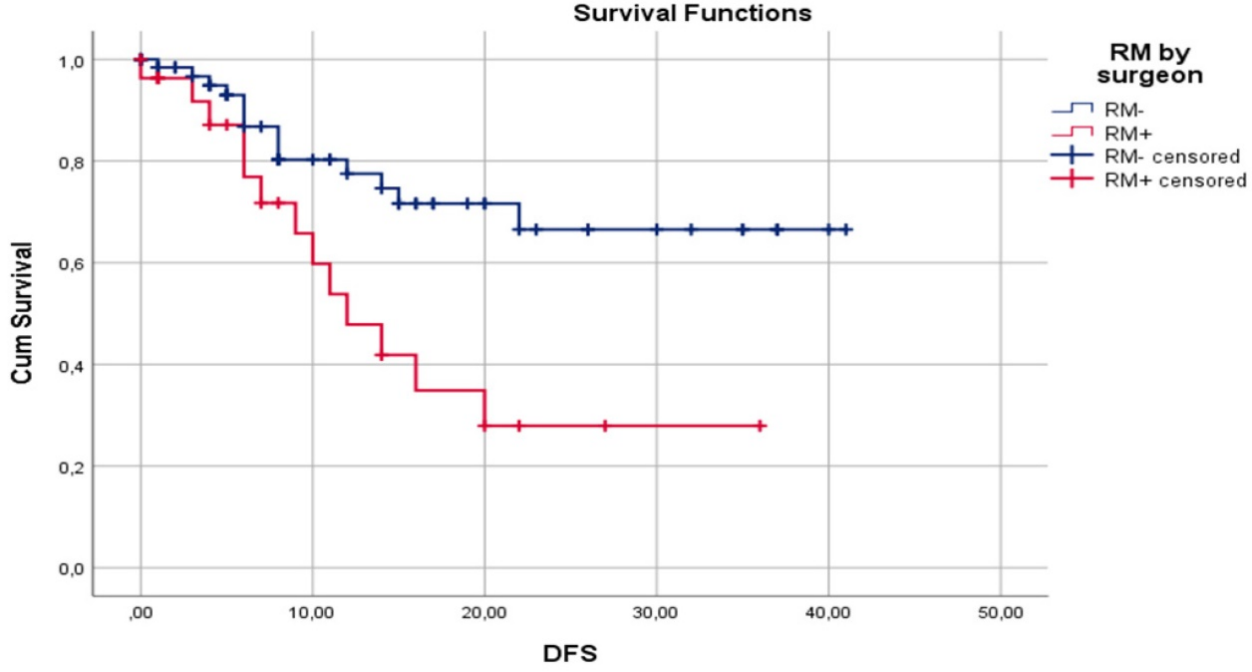
Kaplan-Meier curves for DFS in relation to surgeon assessed liver RM.

**Figure 2:**
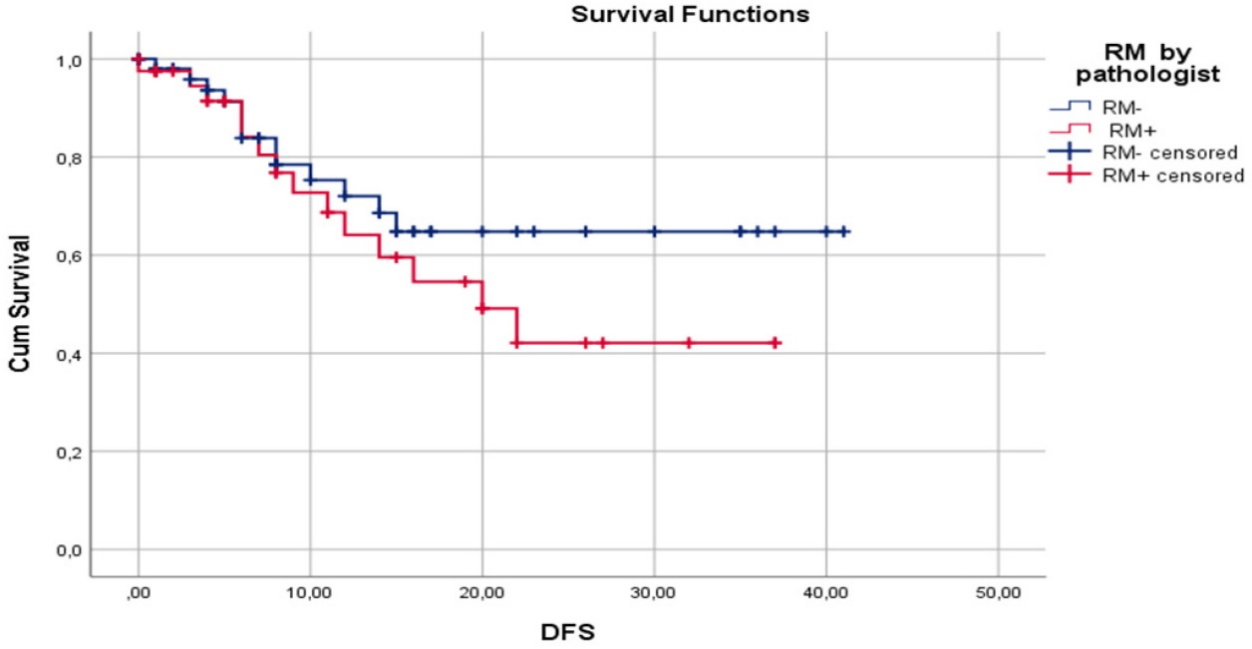
Kaplan-Meier curves for DFS in relation to pathologist assessed liver RM.

Median DFS for surgeon assessed RM- and RM+ were 30.7±2.33 and 17.2±2.98 months, respectively (p=0.02). Median DFS for pathologist assessed RM- and RM+ were 29.5±2.63 and 22.2±2.75 months, respectively (p= 0.47). One-year DFS for surgeon assessed RM- and RM+ were 77.5% and 47.8%, respectively (p=0.01). One-year DFS for pathologist assessed RM- and RM+ were 72.0% and 64.8%, respectively (p=0.59). Three-year DFS for surgeon assessed RM- and RM+ were 66.5% and 27.9%, respectively (p=0.04). Three-year DFS for pathologist assessed RM- and RM+ were 64.1% and 42.1%, respectively (p=0.11). Disease-free survival by surgeon assessed and pathologist assessed CRLM resection margin are shown in **Figure 1** and** Figure 2**, respectively.

## Discussion

The goal of surgical treatment of CRLM is to achieve R0 resection, no identifiable tumor cells on or within 1mm from the inked resection margin. Despite this, there is currently no established standard for intraoperative assessment of RM after liver resection for CRLM. According to our knowledge, there is no published data about the accuracy and reliability of standard intraoperative assessment of RM. In this prospective study, we found that surgical assessment of RM of the RLS, when compared to the “gold standard” histopathological assessment, had good specificity (94.6%) and unacceptable sensitivity (61.5%). These Sp and Sn values were even worse when analyzed on a per patient basis, 91.7% and 55.3%, respectively. Perfect agreement of assessment of RM between surgeon and pathologist should be an ideal scenario, but it proved to be moderate in our study (Kappa value 0.606 per RLS and 0.498 per patient). This surgeon assessed versus pathologist assessed RM disagreement may impact clinical outcome. Hence, we analyzed the influence of surgeon assessed versus pathologist assessed RM on the rate of disease recurrence and DFS.

In our study, surgeon assessed RM+ was a significantly better predictor of overall recurrence rate than pathologist assessed RM+, 57.7% (p=0.001) vs. 42.1% (p=0.12), respectively. Moreover, surgeon assessed RM+ was a better predictor of 1- and 3-year DFS. Median DFS for surgeon assessed resection margin was 30.7±2.33 and 17.2±2.98 months for RM- and RM+, respectively (p=0.02). Median DFS for pathologist assessed resection margin was 29.5±2.63 and 22.2±2.75 months, respectively, for RM- and RM+ (p=0.47).

The major challenge in oncological surgery is to make the distinction between tumor and normal tissue intraoperatively. The current standard practice is based on visual assessment and palpation of the tumor during operation 43. IOUS, in addition to palpation and visualization, is a standard for detection and determination of tumor margin for liver resection 44,47,48. It would be an ideal surgical approach to remove the tumor completely, with minimal safety margins, if surgeons could observe precise tumor margins during the operation. There are attempts to implement different imaging techniques for real-time intraoperative mapping and determination of hepatic tumors 42,43,49-51. Near-infrared fluorescence imaging (NIR) with indocyanine green (ICG) is the most tested experimental technique for identification and demarcation of liver lesions. It was first demonstrated by Ishizawa et al. that, several hours or days after administration, ICG was present in hepatocellular carcinoma and in the rim of liver parenchyma around CRLM 52. This intra-operative imaging technique is useful for detection of the lesion, although it can only be visible to a depth of 1cm within the liver parenchyma. Van Der Vorst et al. detected additional small and superficially located CRLM using NIR fluorescence in 5 of 40 patients (12.5%); these lesions were undetectable by preoperative computed tomography, intraoperative ultrasound, visual inspection, and palpation 51. Peloso et al. in 25 consecutive patients revealed with NIR camera plus ICG a total of 77 metastatic liver nodules. Preoperative CT demonstrated 45 (58.4%) and IOUS showed 55 (71.4%). Preoperative CT and IOUS alone were inferior to the combined use of Photodynamic Eye (PDE) + ICG and IOUS in the detection of lesions of ≤3 mm in size 40. Marino et al. analyzed the impact of NIR with ICG on 40 patients (55% CRLM, 35% hepatocellular carcinoma) who had robotic-assisted liver resection for malignancy 53. Through IOUS and white-light exploration of the liver surface 43 lesions were detected, whereas with NIR and ICG 52 lesions of the liver surface were identified, including two superficial colorectal metastases missed at the IOUS. The R0 resection rate was 100%, and the mean resection margin was 12 mm. One- and 2-year DFS were 77.2% and 65%, respectively, and 1-year and 2-year overall survival rates were 91% and 84%, respectively 53. Using of ICG fluorescence for real-time assessment of RM during laparoscopic and robot-assisted resection for CRLM was analyzed by Achterberg et al. on 16 CRLM (R0 8 cases and R1 8 cases) with no protruding rim in the wound bed *in vivo* were diagnosed as having a tumor-negative margin in 88% of cases 54. Aoki et al. also used NIR fluorescence with ICG for determination of RM on 25 cases with liver malignancy (12 CRLM and 13 hepatocellular carcinoma). All 30 lesions were resected with tumor free margin (R0) with average RM of 5.4 mm (between 1 and 20 mm) 45. In this study, inclusion criteria included lesions that were located less than 1cm from the liver surface, with no invasion of major vessels, no thrombus in the vessels and no biliary reconstruction was required. This implies that a major disadvantage of such an image-based approach is that it could be applied only on the lesions located no more than 1cm from liver surface. It seems that the major advantage of this method is to detect more lesions, specifically if it is used with standard of care (IOUS, visual inspection and palpation). But, in spite of more resected lesions, there is no evidence of benefit in oncological outcome, including overall survival 55. It is interesting that, despite the continual introductions of new technologies in our daily practice, we still do not know the value and clinical significance of the current standard of care. In spite of the fact that every RM is assessed twice (once by surgeon and once by pathologist), according to our knowledge, all authors in peer-reviewed publications analyzed RM only according to histopathological assessment as a standard of care. Definition of negative resection margin was 10 mm in the past 15-17, 56. Then it was decreased to 1 mm and this value is currently the most accepted definition 28,57-61. But there are many authors who recommend that 0 mm should be the defining criterion for RM+ 31,36,62. In this study, we applied the definition of 1 mm or more for RM-.

The rate of RM+ for patients with resected CRLM is usually reported between 20 and 25% 39,63-65, but with wide variation among published studies. Brudvik et al. published a rate of RM+ of 7.6% 66. Welsh et al. published 8.8%, but Takamoto et al. had a much higher rate of RM+ of 69.4% 59,67. Kuo et al. had even 80.5% of RM+ when less than 10 mm was used as a definition of RM+ and 18.9% when 'involved margin' was used as the criterion for RM+ 68. In our series, RM+ rate per patient was 26.5% as assessed by surgeon and 38.8% according to pathologist assessment. We also calculated RM+ rate per RLS and found RM+ result in 18.7% surgeon assessed and 23.7% for pathologist assessed CRLM resection margin.

There are several recognized risk factors for occurrence of RM+, such as the size of the metastatic liver lesion (in particular, more than 5 cm in diameter), the presence of multiple liver lesions, bilobar liver involvement, duration of operation for CRLM, the presence of synchronous metastases, repeat hepatectomy (re-operative liver surgery), among others 31,33. In a recent study of 3,387 patients from 9 high-volume referral centers across Europe, the type of resection (non-anatomic and anatomic/non-anatomic), the number of liver tumors and the size of tumor were identified as negative risk factors for both open and laparoscopic liver resection, while operative blood loss was an additional risk factor in the laparoscopic liver resection group 69. In studies with smaller number of patients, there is no apparent association between these prognostic factors and RM+ 70-72. There was only borderline statistical significance identified for tumor size and surgeon assessed RM in our study, 29.1 vs. 39.4 mm for RM- vs RM+, respectively (p=0.055). Molecular markers of poor tumor biology are also negative prognostic factors for RM+. KRAS mutation is a negative prognostic factor for RM+, so some authors recommend wider RM or anatomical resection rather than parenchymal-sparing surgery for KRAS+ CRLM 26,31,66,73. Gagniere et al. recommend liver resection for BRAF mutated patients, but only in highly selected patients with node negative primary tumor, CEA < 200 mg/L, and Clinical Risk Score < 4 74. We did not identify a similar molecular marker distribution in our series, likely due to the small number of molecular analyses performed.

Resection margin is a well-known prognostic factor for clinical outcome in patients undergoing resection of CRLM, but it seems that its influence is not consistent across studies and it remains a controversial topic in the oncology community. Many authors have concluded that RM+ is a negative prognostic factor for DFS 22,64,75,76, while others concluded that there is no influence of RM+ upon DFS 23,70,77. Some authors have concluded that RM+ did not have influence on 1-year DFS, but it appeared to have influence on 3- and 5-year DFS 58,59,75. Influence of RM+ on OS is also inconsistent in the literature. There is no influence of RM+ on 1-year OS, but it appears to influence 3- and 5-year OS 60,64,73,75,78. However, some authors have not reported any significant impact of RM+ on OS 39,57,58,70.

There are several potential explanations for this inconsistency across published studies. Vigano et al. concluded that, in the case of detachment of a lesion from the vascular structures, R1 resection on the vascular surface did not significantly impact OS, while R1 resection on the parenchymal margin was an independent negative prognostic factor for OS 29. Neoadjuvant systemic therapy in combination with liver resection does have influence on DFS and OS 80. It was found that neoadjuvant therapy may affect significance of RM+. Andreou et al. analyzed 378 patients treated with pre-operative chemotherapy and concluded that RM+ and minor pathological response to chemotherapy were independent prognostic factors for poor survival. However, they noted that there was no significant difference in 5-year survival between R0 and R1 resection for patients who had optimal or major response to pre-operative chemotherapy 28. This diminished influence of neoadjuvant therapy on RM+ was noticed by others 36,75. Laurent et al. concluded that patients with R1 had increased likelihood of liver recurrence, but R1 liver RM does not significantly impact OS 80. On the other hand, Pandanaboyana et al. concluded that neoadjuvant therapy in R1 patients did not have significant influence on OS and that R1 is a negative prognostic factor for clinical outcome 22. Liu et al. performed a meta-analysis and concluded that there is no influence of pre-operative therapy with FOLFOX and FOLFIRI on survival after R1 resection 32. Margonis et al. concluded that only additional biological therapy (bevacizumab) to the systemic chemotherapy diminished negative influence of R1 resection on OS 81,82.

In our study, cancer recurrence rate by pathologist assessment of RM was 26.7% (16/60) for RM- and 42.1% (16/38) for RM+ cases. This difference may be clinically meaningful, but it is not statistically significant (p=0.115). Also, when comparing RM- and RM+ pathologist assessed cases, median DFS of 29.5±2.63 and 22.2±2.75 months (p=0.47), 1-year DFS of 72.0% and 64.8% (p=0.59), and 3-year DFS of 64.1% and 42.1% (p=0.106), respectively, no statistically significant difference was identified. Reasons for this lack of influence of pathologist assessed RM on clinical outcome could be explained by the high rate of applied chemotherapy (86.7%) and biological therapy (57.8%). A second reason could be fact that the median follow-up period is 16.7 months, but a negative influence of RM+ on DFS and OS are shown later in the clinical course of this disease 82. On the other hand, surgeon assessed RM had statistically significant influence on recurrence rate, 22.1% and 57.7% for RM- and RM+, respectively (p=0.01). Also, surgeon assessment of RM had statistically significant influence on median DFS (30.7±2.33 vs 17.2. ±2.98 months; p=0.02), 1-year DFS (77.5% vs 47.8%; p=0.01), and 3-year DFS (66.5% vs 27.9%; p=0.04) for RM- and RM+, respectively. According to our knowledge, there is no published paper with analysis of influence of standard surgeon assessment of RM on clinical outcome after liver resection for CRLM.

Liver resection is the most potentially curable treatment for patients with CRLM and achieving RM- is the primary aim of surgical resection. However, there is no existing criteria establishing how to assess RM during operation; this is based on subjective RM estimation by the surgeon. Despite this, we tried to define criteria for intra-operative definition of RM+, recognizing that it remains a subjective assessment that may vary among surgeons. Importantly, in the current study we tried to minimize this variation using intraoperative data obtained from one experienced hepato-biliary-pancreatic surgeon. Short follow-up duration is a recognized limitation in this study. Our next step forward will be to include data with longer period of follow-up and to include more surgeons with varying degrees of HBP surgery experience in the study to assess the potential of broadly disseminating this approach across surgical practice settings in the care of patients with CRLM undergoing liver resection.

## Conclusion

There is moderate agreement between surgeon assessed and pathologist assessed RM of CRLM. Specificity is high, while sensitivity is unsatisfactory for surgeon assessed RM+ when pathologist assessment of RM status is used as a “gold standard”. Surgical assessment of RM is a better prognostic factor for recurrence rate and DFS than pathologist assessed RM.

## Figures and Tables

**Figure A:**
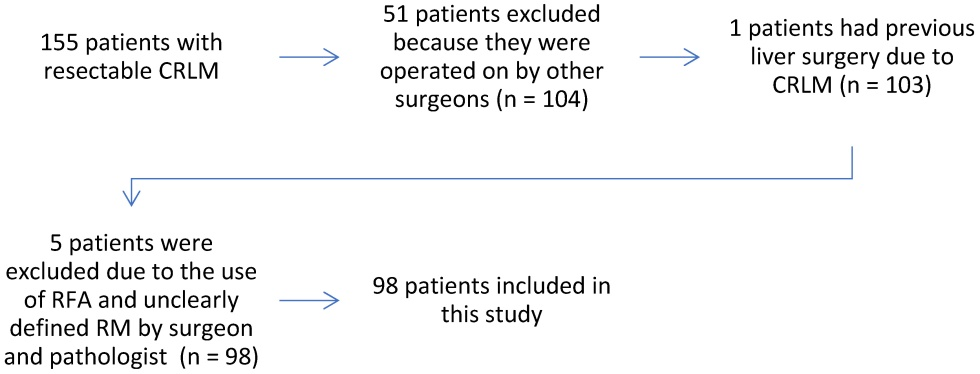
Flowchart of the study.

**Table 1:** Characteristics of patients with CRLM

	SURGEON					PATHOLOGIST					TOTAL	
	RM-		RM+		*p*	RM-		RM+		*P*	No	%
	No	%	No	%		No	%	No	%			
**Sex**												
Male	45	73.8	16	26.2	0.932	35	57.4	26	42.6	0.321	61	62.2
Female	27	73.0	10	27.0		25	67.6	12	32.4		37	37.8
**Age (y)**												
Male	64.1 ± 8.64		63.0 ± 7.55		0.651	63.7 ± 8.92		63.9 ± 7.61		0.910	63.8 ± 8.32	
Female	62.1 ± 12.4		53.6 ± 9.92		0.060	62.7 ± 11.9		53.7 ± 10.9		0.036	59.8 ± 12.2	
**Location**												
Right	16	84.2	3	15.8	0.242	15	78.9	4	21.1	0.079	19	19.4
Left	56	70.9	23	29.1		45	57.0	34	43.0		79	80.6
**Time**												
Synchronous	35	70.0	15	30.0	0.432	31	62.0	19	38.0	0.874	48	49.0
Metachronous	37	77.1	11	22.9		29	60.4	19	39.6		50	51.0
**T**												
0	1	100	0	0	0.091	1	100.0	0	0	0.444	1	1.1
1	1	100	0	0		0	0	1	100		1	1.1
2	4	80.0	1	20.0		3	60.0	2	40.0		5	5.1
3	56	74.7	19	25.3		48	64.0	27	36.0		75	76.3
4	5	50.0	5	50.0		4	40.0	6	60.0		10	10.2
x	5	83.3	1	16.7		4	66.7	2	33.3		6	6.2
**N**												
0	22	84.6	4	15.4	0.460	19	73.1	7	26.9	0.372	26	26.5
1	23	62.2	14	37.8		20	54.1	17	45.9		37	37.8
2	21	75.0	7	25.0		17	60.7	11	39.3		28	28.6
x	5	83.3	1	16.7		4	66.7	2	33.3		6	7.1
**G**												
1	7	87.5	1	12.5	0.883	7	87.5	1	12.5	0.211	8	8.2
2	44	72.1	17	27.9		36	59.0	25	41.0		61	62.2
3	6	85.7	1	14.3		4	57.1	3	42.9		7	7.1
x	15	68.2	7	31.8		13	59.1	9	40.9		22	22.4
**LVI**												
Present	47	69.1	21	30.9	0.145	40	58.8	28	41.2	0.468	68	69.4
Absent	25	83.3	5	16.7		20	66.7	10	33.3		30	30.6
**PNI**												
Present	29	76.3	9	23.7	0.616	23	60.5	15	39.5	0.911	38	38.8
Absent	43	71.7	17	28.3		37	61.7	23	38.3		60	61.2
**LN**												
Examined	15.9 ± 8.26		16.3 ± 7.24		0.828	16.1 ± 8.59		15.8 ± 6.93		0.857	16.0 ± 7.96	
Positive	2.8 ± 3.96		2.8 ± 2.65		0.996	2.5 ± 3.17		3.5 ± 4.26		0.202	2.8 ± 3.64	
**Urgency**												
Yes	17	89.5	2	10.5	0.08	13	68.4	6	31.6	0.478	19	19.4
No	55	69.6	24	30.4		47	59.5	32	40.5		79	80.6
**Diametar (mm), NMI**	29.1 ± 17.9		39.4 ± 22.6		0.055	29.3 ±18.8		35.5 ± 20.6		0.201	32.0 ± 19.7	
**Resection technique**												
Combined	50	73.5	18	26.6	0.719	45	66.2	23	33.8	0.092	68	69.4
Ultracision	22	75.9	7	24.1		15	51.7	14	48.3		29	29.6
Hydrojet	0	0.0	1	100.0		0	0.0	1	100.0		1	1.0
**Type of resection**												
Anatomical	40	72.7	15	27.3	0.853	35	63.6	20	36.4	0.584	55	56.1
Non-anatomical	32	74.4	11	25.6		25	58.1	18	41.9		43	43.9
**Pringle manevar**												
Present	23	63.9	13	36.1	0.104	22	61.1	14	38.9	0.884	36	36.7
Absent	49	79.0	13	21.0		38	61.3	24	38.7		62	63.3
**Macroscopic incompleteness**												
Yes	2	22.2	7	77.8	0.00	2	22.2	7	77.8	0.011	9	9.2
No	70	78.6	19	21.4		58	65.2	31	34.8		89	90.8
**Diametar (mm), pathology**	31.5 ± 24.7		38.5 ± 29.6		0.256	32.8 ± 26.5		34.1 ± 25.9		0.813	33.3 ± 26.1	
**Kras**												
Wild	13	61.9	8	38.1	0.937	11	52.4	10	47.6	0.528	21	21.4
Mutated	12	63.2	7	36.8		8	42.1	11	57.9		19	19.4
**HT (preoperative)**												
I-VI	7	70.0	3	30.0	0.547	6	60.0	4	40.0	0.488	10	31.3
VII-XII	12	66.7	6	33.3		11	61.1	7	38.9		18	56.2
>XII	2	50.0	2	50.0		1	25.0	3	75.0		4	12.5
**HT (preoperative)**												
HT only	10	76.9	3	23.1	0.280	9	69.2	4	30.8	0.234	13	40.6
HT + Biological TH	11	57.9	8	42.1		9	47.4	10	52.6		19	59.4
**HT (perioperative)**												
I-VI	3	75.0	1	25.0	0.126	1	25.0	3	75.0	0.640	4	8.9
VII-XII	19	86.4	3	13.6		15	68.2	7	31.8		22	48.9
>XII	12	63.2	7	36.8		13	68.4	6	31.6		19	42.2
**HT (perioperative)**												
HT only	13	68.4	6	31.6	0.352	14	73.7	5	26.3	0.279	19	42.2
HT + Biological TH	21	80.8	5	19.2		15	57.7	11	42.3		26	57.8
**Time Dg CRC-Dg CRLM (m)**	11.0 ± 17.9		6.4 ± 11.6		0.221	11.4 ± 19.1		7.23 ± 11.0		0.221	9.8 ± 16.5	
**Time Dg-Op CRLM (m)**	6.2 ± 4.79		8.2 ± 6.09		0.094	6.8 ± 6.09		6.6 ± 5.87		0.858	6.7 ± 5.21	
**Time Op CRC-Op CRLM (m)**	14.8 ± 17.9		12.9 ± 17.2		0.631	15.8 ± 19.3		11.9 ± 14.6		0.292	14.3 ± 17.7	
**Recurrence rate**												
Present	17	53.1	15	46.9	0.01	16	50.0	16	50.0	0.115	32	32.7
Absent	55	83.3	11	16.7		44	66.7	22	33.3		66	67.3
**Relaps of the liver**												
Present	14	51.9	13	48.1	0.05	13	48.1	14	51.9	0.174	27	27.6
Absent	57	80.3	14	19.7		45	63.4	26	36.6		71	72.4
**Recurrence of others**												
Present	3	60.0	2	40.0	0.489	3	60.0	2	40.0	0.955	5	5.1
Absent	69	74.2	24	25.8		57	61.3	36	38.7		93	94.9
**Status**												
No evidence of disease	21	80.8	5	19.2	0.398	21	80.8	5	19.2	0.077	26	26.5
Alive with disease	38	69.1	17	30.9		27	49.1	28	50.9		55	56.1
Dead	12	70.6	5	29.4		10	58.8	7	41.2		17	17.3
**DFS (m)**	30.7± 2.33		17.2 ± 2.98		0.021	29.5 ± 2.63		22.2 ± 2.75		0.473	26.9 ± 2.09	

**Table 2:** Agreement of pathologist assessed and surgeon assessed RM+ by RLS

RM+	Pathologist		
	Yes	No	
**Surgeon**			
Yes	32 (78.0%)	9 (22.0%)	41 (18.7%)
No	20 (11.2%)	158 (88.8%)	178 (81.3%)
	52 (23.7%)	167 (76.3%)	219 (100%)
		**No.**	**Percentage, %**
Sensitivity		32/52	61.5%
Specificity		158/167	94.6%
Positive predictive value		32/41	78.0%
Negative predictive value		158/178	88.8%
Total accuracy		(158+32)/219	86.8%
Mc Nemar			p=0.061
Cohen kappa		0.606	p=0.000

**Table 3:** Agreement of pathologist assessed and surgeon assessed RM+ by patient

RM+	Pathologist		
	Yes	No	
**Surgeon**			
Yes	21 (80.8%)	5 (19.2%)	26 (26.5%)
No	17 (23.6%)	55 (76.4%)	72 (73.5%)
	38 (38.8%)	60 (61.2%)	98 (100%)
		**No.**	**Percentage, %**
Sensitivity		21/38	55.3
Specificity		55/60	91.7
Positive predictive value		21/26	80.8
Negative predictive value		55/72	76.4
Total accuracy		(55+21)/98	77.6
Mc Nemar			p=0.017
Cohen kappa		0.498	p=0.000

## Abbreviations

CRC: colorectal cancer
CRLM: colorectal liver metastases
DFS: disease-free survival
HPA: Histopathological assessment
ICG: Indocyanine green
IOUS: Intra-operative ultrasonography
MDT: multi-disciplinary team
NIR: Near-infrared fluorescence imaging
RM: resection margin
RM-: resection margin of 1 mm or more
RM+: resection margin less than 1 mm
R0: no identifiable tumor cells on or within 1 mm from the inked resection margin
RLS: resected liver specimen
SA: surgeon assessment
SOC: standard of care

## References

[1] Bray F, Ferlay J, Soerjomataram I (2018). Global cancer statistics 2018: GLOBOCAN estimates of incidence and mortality worldwide for 36 cancers in 185 countries. CA Cancer J Clin.

[2] Coppa GF (1990). Surgical resection for colorectal hepatic metastases. Bull N Y Acad Med.

[3] Valderrama-Treviño AI, Barrera-Mera B, Ceballos-Villalva JC (2017). Hepatic Metastasis from Colorectal Cancer. Euroasian J Hepatogastroenterol.

[4] Adam R, Kitano Y (2019). Multidisciplinary approach of liver metastases from colorectal cancer. Ann Gastroenterol Surg.

[5] Akgül Ö, Çetinkaya E, Ersöz Ş (2014). Role of surgery in colorectal cancer liver metastases. World J Gastroenterol.

[6] Bouviez N, Lakkis Z, Lubrano J (2014). Liver resection for colorectal metastases: results and prognostic factors with 10-year follow-up. Langenbecks Arch Surg.

[7] Chow FC, Chok KS (2019). Colorectal liver metastases: An update on multidisciplinary approach. World J Hepatol.

[8] de Ridder JA, Lemmens VE, Overbeek LI (2016). Liver resection for metastatic disease; A population-based analysis of trends. Dig Surg.

[9] Dexiang Z, Li R, Ye W (2012). Outcome of Patients with Colorectal Liver Metastasis: Analysis of 1,613 Consecutive Cases. Ann Surg Oncol.

[10] Engstrand J, Nilsson H, Strömberg C (2018). Colorectal cancer liver metastases - a population-based study on incidence, management and survival. BMC Cancer.

[11] Imai K, Adam R, Baba H (2019). How to increase the resectability of initially unresectable colorectal liver metastases: A surgical perspective. Ann Gastroenterol Surg.

[12] Vera R, González-Flores E, Rubio C (2020). Multidisciplinary management of liver metastases in patients with colorectal cancer: a consensus of SEOM, AEC, SEOR, SERVEI, and SEMNIM. Clin Transl Oncol.

[13] Xu F, Tang B, Jin TQ (2018). Current status of surgical treatment of colorecatl liver metastases. World J Clin Cases.

[14] Solaini L, Gardini A, Passardi A (2019). Preoperative chemotherapy and resection margin status in colorectal liver metastasis patients: A propensity score-matched analysis. Am Surg.

[15] Holm A, Bradley E, Aldrete JS (1989). Hepatic Resection of Metastasis from Colorectal Carcinoma Morbidity, Mortality, and Pattern of Recurrence. Ann Surg.

[16] August DA, Sugarbaker PH, Ottow RT (1985). Hepatic Resection of Colorectal Metastases Influence of Clinical Factors and Adjuvant Intraperitoneal 5-Fluorouracil via Tenckhoff Catheter on Survival. Ann Surg.

[17] Ekberg H, Tranberg KG, Andersson R (1986). Determinants of survival in liver resection for colorectal secondaries. Br J Surg.

[18] Kokudo N, Miki Y, Sugai S (2002). Genetic and histological assessment of surgical margins in resected liver metastases from colorectal carcinoma: minimum surgical margins for successful resection. Arch Surg.

[19] Tsim N, Healey AJ, Frampton AE (2011). Two-Stage Resection for Bilobar Colorectal Liver Metastases: R0 Resection Is the Key. Ann Surg Oncol.

[20] Konopke R, Kersting S, Makowiec F (2008). Resection of Colorectal Liver Metastases: Is a Resection Margin of 3 mm Enough?. World J Surg.

[21] de Haas RJ, Wicherts DA, Flores E (2008). R1 Resection by Necessity for Colorectal Liver Metastases: is it still a contraindication to surgery. Ann Surg.

[22] Pandanaboyana S, White A, Pathak S (2015). Impact of Margin Status and Neoadjuvant Chemotherapy on Survival, Recurrence After Liver Resection for Colorectal Liver Metastasis. Ann Surg Oncol.

[23] Montalti R, Tomassini F, Laurent S (2015). Impact of surgical margins on overall and recurrence-free survival in parenchymal-sparing laparoscopic liver resections of colorectal metastases. Surg Endosc.

[24] Martínez-Cecilia D, Wicherts DA, Cipriani F (2021). Impact of resection margins for colorectal liver metastases in laparoscopic and open liver resection: a propensity score analysis. Surg Endosc.

[25] Pandanaboyana S, Bell R, White A (2018). Impact of parenchymal preserving surgery on survival and recurrence after liver resection for colorectal liver metastasis. ANZ J Surg.

[26] Margonis GA, Sasaki K, Andreatos N (2017). KRAS Mutation Status Dictates Optimal Surgical Margin Width in Patients Undergoing Resection of Colorectal Liver Metastases. Ann Surg.

[27] Angelsen JH, Viste A, Løes IM (2015). Predictive factors for time to recurrence, treatment and post-recurrence survival in patients with initially resected colorectal liver metastases. World J Surg Oncol.

[28] Andreou A, Aloia TA, Brouquet A (2013). Margin status remains an important determinant of survival after surgical resection of colorectal liver metastases in the era of modern chemotherapy. Ann Surg.

[29] Viganò L, Procopio F, Cimino MM (2016). Is Tumor Detachment from Vascular Structures Equivalent to R0 Resection in Surgery for Colorectal Liver Metastases? An Observational Cohort. Ann Surg Oncol.

[30] Oshi M, Margonis GA, Sawada Y (2019). Higher Tumor Burden Neutralizes Negative Margin Status in Hepatectomy for Colorectal Cancer Liver Metastasis. Ann Surg Oncol.

[31] Sadot E, Groot Koerkamp B, Leal JN (2015). Resection Margin and Survival in 2368 Patients Undergoing Hepatic Resection for Metastatic Colorectal Cancer: Surgical Technique or Biologic Surrogate?. Ann Surg.

[32] Liu W, Sun Y, Zhang L (2015). Negative surgical margin improved long-term survival of colorectal cancer liver metastases after hepatic resection: a systematic review and meta-analysis. Int J Colorectal Dis.

[33] Memeo R, de Blasi V, Adam R (2018). Margin Status is Still an Important Prognostic Factor in Hepatectomies for Colorectal Liver Metastases: A Propensity Score Matching Analysis. World J Surg.

[34] Tranchart H, Chirica M, Faron M (2013). Prognostic impact of positive surgical margins after resection of colorectal cancer liver metastases: reappraisal in the era of modern chemotherapy. World J Surg.

[35] Paniccia A, Schulick RD (2016). Surgical Margin in Hepatic Resections for Colorectal Metastasis: Should We Care?. Curr Colorectal Cancer Rep.

[36] Ayez N, Lalmahomed ZS, Eggermont AM (2012). Outcome of Microscopic Incomplete Resection (R1) of Colorectal Liver Metastases in the Era of Neoadjuvant Chemotherapy. Ann Surg Oncol.

[37] Viganò L, Costa G, Cimino MM (2018). R1 Resection for Colorectal Liver Metastases: a Survey Questioning Surgeons about Its Incidence, Clinical Impact, and Management. J Gastrointest Surg.

[38] Creasy JM, Sadot E, Koerkamp BG (2018). Actual 10-year survival after hepatic resection of colorectal liver metastases: what factors preclude cure?. Surgery.

[39] Postriganova N, Kazaryan AM, Røsok BI (2014). Margin status after laparoscopic resection of colorectal liver metastases: Does a narrow resection margin have an influence on survival and local recurrence?. HPB (Oxford).

[40] Peloso A, Franchi E, Canepa MC (2013). Combined use of intraoperative ultrasound and indocyanine green fluorescence imaging to detect liver metastases from colorectal cancer. HPB (Oxford).

[41] Gao RW, Teraphongphom NT, van den Berg NS (2018). Determination of tumor margins with surgical specimen mapping using near-infrared fluorescence. Cancer Res.

[42] Holt D, Okusanya O, Judy R (2014). Intraoperative near-infrared imaging can distinguish cancer from normal tissue but not inflammation. PLoS One.

[43] Orbay H, Bean J, Zhang Y (2013). Intraoperative Targeted Optical Imaging: A Guide towards Tumor-Free Margins in Cancer Surgery. Curr Pharm Biotechnol.

[44] Hata S, Imamura H, Aoki T (2011). Value of Visual Inspection, Bimanual Palpation, and Intraoperative Ultrasonography During Hepatic Resection for Liver Metastases of Colorectal Carcinoma. World J Surg.

[45] Aoki T, Murakami M, Koizumi T (2018). Determination of the surgical margin in laparoscopic liver resections using infrared indocyanine green fluorescence. Langenbecks Arch Surg.

[46] McHugh ML (2012). Interrater reliability: The kappa statistic. Biochem Medic(Zagreb).

[47] Knol JA, Marn CS, Francis IR (1993). Comparisons of dynamic infusion and delayed computed tomography, intraoperative ultrasound, and palpation in the diagnosis of liver metastases. Am J Surg.

[48] Hoch G, Croise-Laurent V, Germain A (2015). Is intraoperative ultrasound still useful for the detection of colorectal cancer liver metastases?. HPB (Oxford).

[49] Judy RP, Keating JJ, DeJesus EM (2015). Quantification of tumor fluorescence during intraoperative optical cancer imaging. Sci Rep.

[50] Vahrmeijer AL, Hutteman M, van der Vorst JR (2013). Image-guided cancer surgery using near-infrared fluorescence. Nat Rev Clin Oncol.

[51] van der Vorst JR, Schaafsma BE, Hutteman M (2013). Near-infrared fluorescence-guided resection of colorectal liver metastases. Cancer.

[52] Ishizawa T, Fukushima N, Shibahara J (2009). Real-time identification of liver cancers by using indocyanine green fluorescent imaging. Cancer.

[53] Marino MV, Podda M, Fernandez CC (2020). The application of indocyanine green-fluorescence imaging during robotic-assisted liver resection for malignant tumors: a single-arm feasibility cohort study. HPB (Oxford).

[54] Achterberg FB, Sibinga Mulder BG, Meijer RPJ (2020). Real-time surgical margin assessment using ICG-fluorescence during laparoscopic and robot-assisted resections of colorectal liver metastases. Ann Transl Med.

[55] Handgraaf HJM, Boogerd LSF, Höppener DJ (2017). Long-term follow-up after near-infrared fluorescence-guided resection of colorectal liver metastases: A retrospective multicenter analysis. Eur J Surg Oncol.

[56] Iwatsuki S, Dvorchik I, Madariaga JR (1999). Hepatic resection for metastatic colorectal adenocarcinoma: A proposal of a prognostic scoring system. J Am Coll Surg.

[57] Homayounfar K, Liersch T, Niessner M (2010). Multimodal treatment options for bilobar colorectal liver metastases. Langenbecks Arch Surg.

[58] Margonis GA, Spolverato G, Kim Y (2015). Intraoperative Surgical Margin Re-resection for Colorectal Liver Metastasis: Is It Worth the Effort?. J Gastrointest Surg.

[59] Takamoto T, Sugawara Y, Hashimoto T (2016). Two-dimensional assessment of submillimeter cancer-free margin area in colorectal liver metastases. Medicine (Baltimore).

[60] Sasaki K, Margonis GA, Maitani K (2017). The Prognostic Impact of Determining Resection Margin Status for Multiple Colorectal Metastases According to the Margin of the Largest Lesion. Ann Surg Oncol.

[61] Hamady ZZ, Lodge JP, Welsh FK (2014). One-millimeter cancer-free margin is curative for colorectal liver metastases: A propensity score case-match approach. Ann Surg.

[62] Yasuno M, Uetake H, Ishiguro M (2019). mFOLFOX6 plus bevacizumab to treat liver-only metastases of colorectal cancer that are unsuitable for upfront resection (TRICC0808): a multicenter phase II trial comprising the final analysis for survival. Int J Clin Oncol.

[63] Akyuz M, Aucejo F, Quintini C (2016). Factors affecting surgical margin recurrence after hepatectomy for colorectal liver metastases. Gland Surg.

[64] Vandeweyer D, Neo EL, Chen JW (2009). Influence of resection margin on survival in hepatic resections for colorectal liver metastases. HPB (Oxford).

[65] Pulitanò C, Bodingbauer M, Aldrighetti L (2011). Liver resection for colorectal metastases in presence of extrahepatic disease: Results from an international multi-institutional analysis. Ann Surg Oncol.

[66] Brudvik KW, Mise Y, Chung MH (2016). RAS Mutation Predicts Positive Resection Margins and Narrower Resection Margins in Patients Undergoing Resection of Colorectal Liver Metastases. Ann Surg Oncol.

[67] Welsh FK, Tekkis PP, O'Rourke T (2008). Quantificatin of risk of a positive (R1) resection margin following hepatic resection for metastatic colorectal cancer: an aid to clinical decision-making. Surg Oncol.

[68] Kuo IM, Huang SF, Chiang JM (2015). Clinical features and prognosis in hepatectomy for colorectal cancer with centrally located liver metastasis. World J Surg Oncol.

[69] Benedetti Cacciaguerra A, Görgec B Risk Factors of Positive Resection Margin in Laparoscopic and Open Liver Surgery for Colorectal Liver Metastases: A New Perspective in the Perioperative Assessment: A European Multicenter Study. Ann Surg.

[70] Eveno C, Karoui M, Gayat E (2013). Liver resection for colorectal liver metastases with peri-operative chemotherapy: oncological results of R1 resections. HPB (Oxford).

[71] Pencovich N, Houli R, Lubezky N (2019). R1 resection of colorectal liver metastasis - What is the cost of marginal resection?. J Surg Oncol.

[72] Ardito F, Panettieri E, Vellone M (2019). The impact of R1 resection for colorectal liver metastases on local recurrence and overall survival in the era of modern chemotherapy: An analysis of 1,428 resection areas. Surgery.

[73] Brudvik KW, Jones RP, Giuliante F (2019). RAS Mutation Clinical Risk Score to Predict Survival after Resection of Colorectal Liver Metastases. Ann Surg.

[74] Gagnière J, Dupré A, Gholami SS (2020). Is Hepatectomy Justified for BRAF Mutant Colorectal Liver Metastases?. Ann Surg.

[75] Tanaka K, Nojiri K, Kumamoto T (2011). R1 resection for aggressive or advanced colorectal liver metastases is justified in combination with effective prehepatectomy chemotherapy. Eur J Surg Oncol.

[76] Hamada T, Nakai Y, Yasunaga H (2014). Prognostic nomogram for nonresectable pancreatic cancer treated with gemcitabine-based chemotherapy. Br J Cancer.

[77] Di Carlo S, Yeung D, Mills J (2016). Resection margin influences the outcome of patients with bilobar colorectal liver metastases. World J Hepatol.

[78] Mao R, Zhao JJ, Bi XY (2018). Interaction of margin status and tumour burden determines survival after resection of colorectal liver metastases: A retrospective cohort study. Int J Surg.

[79] Khoo E, O'Neill S, Brown E (2016). Systematic review of systemic adjuvant, neoadjuvant and perioperative chemotherapy for resectable colorectal-liver metastases. HPB (Oxford).

[80] Laurent C, Adam JP, Denost Q (2016). Significance of R1 Resection for Advanced Colorectal Liver Metastases in the Era of Modern Effective Chemotherapy. World J Surg.

[81] Margonis GA, Buettner S, Andreatos N (2019). Prognostic Factors Change Over Time After Hepatectomy for Colorectal Liver Metastases. Ann Surg.

[82] Sasaki K, Margonis GA, Andreatos N (2017). Prognostic impact of margin status in liver resections for colorectal metastases after bevacizumab. Br J Surg.

